# Spinal Concussion

**Published:** 1890-06

**Authors:** 


*Spinal Concussion.* By S. N. CLEVENGER, M.D. Pp. 338. Philadelphia and London: F. A. DAVIS. 1889.

There are certain features about this work which strike us as peculiar. Firstly, the author tells us that "the treatises now in use are twenty years behind the times." Now, Herbert Page, of St. Mary's Hospital, in 1881 got the Boylston Prize of Harvard University for his treatise on this very subject; and he has not even been twenty years in the profession: he must be a very *backward* writer! Then our author describes himself as a "member of numerous American scientific and medical societies," while he dates his preface from "Central Music Hall, Chicago." The combination of much science in harmony has long ago been reckoned as music; and we welcome the reappearance of the principle in Chicago. But bad English is not musical; and when on the first page we meet with bad grammar, bad spelling, and wrong quotations, our enthusiasm is somewhat damped. But as we go on our bad impressions are modified, and here and there replaced by good ones.

The work is distinctly worth reading. It is frankly and strongly polemical. The bent of the main arguments is sufficiently shown by his suggestion that a certain symptom group resulting from spinal injury should be known as "Erichsen's Disease." The author has evidently fully studied his subject at the bedside, as well as in books; and if, here and there, his language seems to be somewhat more emphatic than the facts warrant, we cannot but admit that he has a large balance of right on his side. He vigorously attacks and demolishes loose theories and crude statements; and supports his own position with much external aid and considerable internal resource. The work will help to clear away some of the mist in which traumatic spinal pathology is enveloped, and may lead us a little nearer to definiteness and cohesion in our knowledge of a class of injuries which at present is both indefinite and chaotic.

